# Supplementary material for: Do manual therapies have a specific autonomic effect? An overview of systematic reviews
Source: PLoS One. 2021 Dec 2;16(12):e0260642. doi: 10.1371/journal.pone.0260642 (PMC8638932; doi:10.1371/journal.pone.0260642)
Supplement: S1 Table — (DOCX) [file pone.0260642.s002.docx]

S1 Table. Medline search strategy

("Manipulation, Spinal"[Mesh] OR "Manipulation, Osteopathic"[Mesh] OR "Musculoskeletal Manipulations"[Mesh] OR osteopath*[tiab] OR manual therap*[tiab] OR manipulati*[ti] OR manipulative therap*[tiab] OR manipulative techniq*[tiab] OR mobilization*[ti] OR mobilisation*[ti]) AND ("Neurophysiology"[Mesh] OR "Physiology"[Mesh] OR "Autonomic Nervous System Diseases"[Majr] OR "Autonomic Nervous System"[Majr] OR "Sympathetic Nervous System"[Majr] OR "physiology"[sh] OR neurophysiologic*[ti] OR nervous system[ti] OR autonomic nervous[tiab] OR sympathetic nervous[tiab]) AND (systematic[sb] OR review[ti])

1 "Manipulation, Spinal"[Mesh]

2 "Manipulation, Osteopathic"[Mesh]

3 "Musculoskeletal Manipulations"[Mesh]

4 osteopath*[tiab]

5 manual therap*[tiab]

6 manipulati*[ti]

7 manipulative therap*[tiab]

8 manipulative techniq*[tiab]

9 mobilization*[ti]

10 mobilisation*[ti]

11 #1 OR #2 OR #3 OR #4 OR #5 OR #6 OR #7 OR #8 OR #9 OR #10

12 "Neurophysiology"[Mesh]

13 "Physiology"[Mesh]

14 "Autonomic Nervous System Diseases"[Majr]

15 "Autonomic Nervous System"[Majr]

16 "Sympathetic Nervous System"[Majr]

17 "physiology"[sh]

18 neurophysiologic*[ti]

19 nervous system[ti]

20 autonomic nervous[tiab]

21 sympathetic nervous[tiab]

22 #12 OR #13 OR #14 OR #15 OR #16 OR #17 OR #18 OR #19 OR #20 OR #21

23 #11 AND #22

24 systematic[sb] OR review[ti]

25 #23 AND #24
