# Supplementary material for: Do manual therapies have a specific autonomic effect? An overview of systematic reviews
Source: PLoS One. 2021 Dec 2;16(12):e0260642. doi: 10.1371/journal.pone.0260642 (PMC8638932; doi:10.1371/journal.pone.0260642)
Supplement: S3 Table — (DOCX) [file pone.0260642.s004.docx]

S3 Table. Robis assessment of the included reviews.

| **Phase 2: Identifying concerns with the review process** | Systematic Reviews | | | | | | | |  |
| --- | --- | --- | --- | --- | --- | --- | --- | --- | --- |
| **DOMAIN 1: STUDY ELIGIBILITY CRITERIA** | Schmid 2008 | Hegedus 2011 | | Chu 2014 | Kingston 2014 | | Lascurain 2016 | |  |
| 1.1 Did the review adhere to pre-defined objectives and eligibility criteria? | PY | PY | | PY | PY | | PY | |  |
| 1.2 Were the eligibility criteria appropriate for the review question? | Y | PN | | Y | Y | | Y | |  |
| 1.3 Were eligibility criteria unambiguous? | Y | PN | | Y | Y | | PN | |  |
| 1.4 Were any restrictions in eligibility criteria based on study characteristics appropriate (e.g. date, sample size, study quality,outcomes measured)? | Y | PY | | Y | PY | | PY | |  |
| 1.5 Were any restrictions in eligibility criteria based on sources of information appropriate (e.g. publication status or format, language, availability of data)? | Y | PY | | Y | PN | | PN | |  |
| Concerns regarding specification of study eligibility criteria | LOW | UNCLEAR | | LOW | LOW | | UNCLEAR | |  |
| Rationale for concern: |  | not clear what it means effects beyond immediate effects | | | | | Not clear the type of intervention, as the results retrieve only cervical interventions, language and publication types not included, not specified comparators | |  |
| **DOMAIN 2: IDENTIFICATION AND SELECTION OF STUDIES** |  |  | |  |  | |  | |  |
| 2.1 Did the search include an appropriate range of databases/electronic sources for published and unpublished reports? | Y | PY | | Y | Y | | Y | |  |
| 2.2 Were methods additional to database searching used to identify relevant reports? | Y | PY | | PY | N | | PY | |  |
|  |  |  | |  |  | |  | |  |
| 2.3 Were the terms and structure of the search strategy likely to retrieve as many eligible studies as possible? | PY | PN | | PY | NI | | PY | |  |
|  |  |  | |  |  | |  | |  |
| 2.4 Were restrictions based on date, publication format, or language I appropriate? | Y | PY | | PN | PN | | PN | |  |
|  |  |  | |  |  | |  | |  |
| 2.5 Were efforts made to minimise error in selection of studies? |  |  | |  |  | |  | |  |
|  | PY | PY | | PY | PY | | PN | |  |
| Concerns regarding methods used to identify and/or select studies LOW/HIGH/UNCLEAR |  |  | |  |  | |  | |  |
|  | LOW | LOW | | LOW | UNCLEAR | | UNCLEAR | |  |
| Rationale for concern: |  |  | |  |  | | only one reviewer in the selection process, articles not wrote in english were not included | |  |
|  |  |  | |  |  | |  | |  |
| **DOMAIN 3: DATA COLLECTION AND STUDY APPRAISAL** |  |  | |  |  | |  | |  |
| 3.1 Were efforts made to minimise error in data collection? | PY | PY | | PY | PY | | PY | |  |
| 3.2 Were sufficient study characteristics available for both review authors and readers to be able to interpret the results? | PY | PY | | PY | PN | | PY | |  |
| 3.3 Were all relevant study results collected for use in the synthesis? | PY | PY | | Y | Y | | PN | |  |
| 3.4 Was risk of bias (or methodological quality) formally assessed using appropriate criteria? | Y | PY | | Y | Y | | Y | |  |
| 3.5 Were efforts made to minimise error in risk of bias assessment? | PY | PY | | PY | PY | | Y | |  |
| Concerns regarding methods used to collect data and appraise studies LOW/HIGH/UNCLEAR | LOW | LOW | | LOW | LOW | | LOW | |  |
| Rationale for concern: | JADAD SCALE WITH ALLOCATION CONCEALMENT | | | | | | | |  |
| **DOMAIN 4: SYNTHESIS AND FINDINGS** |  |  | |  |  | |  | |  |
| 4.1 Did the synthesis include all studies that it should? | PY | PY | | Y | PY | | PY | |  |
| 4.2 Were all pre-defined analyses reported or departures explained? | NI | N | | PY | NI | | NI | |  |
| 4.3 Was the synthesis appropriate given the nature and similarity in the research questions, study designs and outcomes across included studies? | N | PN | | Y | PY | | N | |  |
| 4.4 Was between-study variation (heterogeneity) minimal or addressed in the synthesis? | N | N | | Y | N | | N | |  |
| 4.5 Were the findings robust, e.g. as demonstrated through funnel plot or sensitivity analyses? | N | N | | Y | N | | N | |  |
| 4.6 Were biases in primary studies minimal or addressed in the synthesis? | PY | Y | | PY | PN | | PY | |  |
| Concerns regarding methods used to collect data and appraise studies | HIGH | UNCLEAR | | LOW | HIGH | | HIGH | |  |
| Rationale for concern: | NO PROTOCOL INFORMATIONTherefore, we could only include three outcome parameters in our statistical analysis. IMPORTANT BETWWEN-STUDY VARIOATION WAS NOT ACCOUNTED FOR | the studies included were asssessing short term effects, not immediate but 5-10 later, not clear if the research question is answered with this studies | | | | | no protocol information, stadistic analyse pre-defined, no tables | |  |
| **Phase 3: Judging risk of bias** |  |  | |  |  | |  | |  |
| Summarize the concerns identified during the Phase 2 assessment: |  |  | |  |  | |  | |  |
| 1. Concerns regarding specification of study eligibility criteria | LOW |  | | LOW |  | |  | |  |
|  |  | UNCLEAR | | | LOW | | UNCLEAR | |  |
| 2. Concerns regarding methods used to identify and/or select studies | LOW |  | | LOW |  | |  | |  |
|  |  | LOW | |  | UNCLEAR | | UNCLEAR | |  |
| 3. Concerns regarding methods used to collect data and appraise studies | LOW |  | | LOW |  | |  | |  |
|  |  | LOW | |  | LOW | | LOW | |  |
| 4. Concerns regarding the synthesis and findings | HIGH |  | | LOW |  | |  | |  |
|  |  | UNCLEAR | | | HIGH | | HIGH | |  |
| **RISK OF BIAS IN THE REVIEW** |  |  | |  |  | |  | |  |
| Describe whether conclusions were supported by the evidence: |  |  | |  |  | |  | |  |
| A. Did the interpretation of findings address all of the concerns identified in Domains 1 to 4? | |  | |  |  | |  | |  |
|  | PY | PY | | Y | PY | | PN | |  |
| B. Was the relevance of identified studies to the review's research question appropriately considered? | |  | |  |  | |  | |  |
|  | PN | PN | | Y | PY | | PN | |  |
| C. Did the reviewers avoid emphasizing results on the basis of their statistical significance? |  |  | |  |  | |  | |  |
|  | PN | PN | | PY | PY | | NI | |  |
| Risk of bias in the review RISK: LOW/HIGH/UNCLEAR |  |  | |  |  | |  | |  |
|  | UNCLEAR | UNCLEAR | | LOW | UNCLEAR | | HIGH | |  |
| Rationale for risk: | 9 OUT OF 15 INCLUDED ANS MEASURES, ONLY 3 OUTCOMES WERE INCLUDED IN THE ANALYSIS BUT REPORTED AND ALSO LIMITATIONS | Same rational than above | | | There is insufficient information reportd to make a judgement on risk of bias | | general research question not retrieving enough articles for each outcome to conclude in a robust way | |  |
|  |  |  | |  |  | |  | |  |
| **Phase 2: Identifying concerns with the review process** | Systematic Reviews | | | | |  | |  | |
| **DOMAIN 1: STUDY ELIGIBILITY CRITERIA** | Amoroso-Borges 2017 | | Galíndez-Ibarbengoetxea 2017 | | | Araujo 2018 | | Navarro-Santana 2019 | |
| 1.1 Did the review adhere to pre-defined objectives and eligibility criteria? | PY | | PY | | | Y | | Y | |
| 1.2 Were the eligibility criteria appropriate for the review question? | Y | | Y | | | Y | | PY | |
| 1.3 Were eligibility criteria unambiguous? | Y | | Y | | | Y | | Y | |
| 1.4 Were any restrictions in eligibility criteria based on study characteristics appropriate (e.g. date, sample size, study quality,outcomes measured)? | Y | | PY | | | Y | | PN | |
| 1.5 Were any restrictions in eligibility criteria based on sources of information appropriate (e.g. publication status or format, language, availability of data)? | PN | | PN | | | PY | | PY | |
| Concerns regarding specification of study eligibility criteria | LOW | | LOW | | | LOW | | UNCLEAR | |
| Rationale for concern: |  | | NOT PROTOCOL INFORMATION, NO ENGLISH, INDEXED STUDIES, | | | | | OUTCOME RESTRICTED TO SC, ST WHEN AIM TO ASSESS SYMPATHETIC NS | |
| **DOMAIN 2: IDENTIFICATION AND SELECTION OF STUDIES** |  | |  | | |  | |  | |
| 2.1 Did the search include an appropriate range of databases/electronic sources for published and unpublished reports? | Y | | PY | | | Y | | Y | |
| 2.2 Were methods additional to database searching used to identify relevant reports? | N | | PN | | | Y | | Y | |
|  |  | |  | | |  | |  | |
| 2.3 Were the terms and structure of the search strategy likely to retrieve as many eligible studies as possible? | NI | | PY | | | Y | | Y | |
|  |  | |  | | |  | |  | |
| 2.4 Were restrictions based on date, publication format, or language I appropriate? | PN | | PN | | | PY | | PY | |
|  |  | |  | | |  | |  | |
| 2.5 Were efforts made to minimise error in selection of studies? |  | |  | | |  | |  | |
|  | PY | | PY | | | Y | | Y | |
| Concerns regarding methods used to identify and/or select studies LOW/HIGH/UNCLEAR |  | |  | | |  | |  | |
|  | UNCLEAR | | UNCLEAR | | | LOW | | LOW | |
| Rationale for concern: | no additional database searching, no information about search string, no protocol or appendix,language and indexed journals | | no protocol or appendix,language and indexed journals | | |  | |  | |
|  |  | |  | | |  | |  | |
| **DOMAIN 3: DATA COLLECTION AND STUDY APPRAISAL** |  | |  | | |  | |  | |
| 3.1 Were efforts made to minimise error in data collection? | PY | | PY | | | Y | | Y | |
| 3.2 Were sufficient study characteristics available for both review authors and readers to be able to interpret the results? | PY | | PN | | | PY | | Y | |
| 3.3 Were all relevant study results collected for use in the synthesis? | PY | | PN | | | PY | | Y | |
| 3.4 Was risk of bias (or methodological quality) formally assessed using appropriate criteria? | PN | | Y | | | Y | | Y | |
| 3.5 Were efforts made to minimise error in risk of bias assessment? | Y | | PY | | | Y | | Y | |
| Concerns regarding methods used to collect data and appraise studies LOW/HIGH/UNCLEAR | UNCLEAR | | UNCLEAR | | | LOW | | LOW | |
| Rationale for concern: | NARRATIVE RESULTS, WITHOUT CI,DATA NOT EXPLICITED IN METHODS | | ONLY 3 ARTICLES FOR AUTONOMIC OUTCOMES RESULTS | | |  | |  | |
| **DOMAIN 4: SYNTHESIS AND FINDINGS** |  | |  | | |  | |  | |
| 4.1 Did the synthesis include all studies that it should? | PY | | PY | | | Y | | Y | |
| 4.2 Were all pre-defined analyses reported or departures explained? | NI | | NI | | | Y | | Y | |
| 4.3 Was the synthesis appropriate given the nature and similarity in the research questions, study designs and outcomes across included studies? | PN | | PN | | | Y | | PY | |
| 4.4 Was between-study variation (heterogeneity) minimal or addressed in the synthesis? | N | | N | | | Y | | Y | |
| 4.5 Were the findings robust, e.g. as demonstrated through funnel plot or sensitivity analyses? | N | | N | | | Y | | PY | |
| 4.6 Were biases in primary studies minimal or addressed in the synthesis? | N | | PY | | | Y | | PY | |
| Concerns regarding methods used to collect data and appraise studies | HIGH | | UNCLEAR | | | LOW | | LOW | |
| Rationale for concern: | NARRATIVE RESULTS, WITHOUT CI, DATA NOT EXPLICITED IN METHODS | | STADISTICAL ANALYSIS POOR DESCRIBED | | |  | |  | |
| **Phase 3: Judging risk of bias** |  | |  | | |  | |  | |
| Summarize the concerns identified during the Phase 2 assessment: |  | |  | | |  | |  | |
| 1. Concerns regarding specification of study eligibility criteria |  | |  | | |  | |  | |
|  | LOW | | LOW | | | LOW | | UNCLEAR | |
| 2. Concerns regarding methods used to identify and/or select studies |  | |  | | |  | |  | |
|  | UNCLEAR | | UNCLEAR | | | LOW | | LOW | |
| 3. Concerns regarding methods used to collect data and appraise studies |  | |  | | |  | |  | |
|  | UNCLEAR | | UNCLEAR | | | LOW | | LOW | |
| 4. Concerns regarding the synthesis and findings |  | |  | | |  | |  | |
|  | HIGH | | UNCLEAR | | | LOW | | LOW | |
| **RISK OF BIAS IN THE REVIEW** |  | |  | | |  | |  | |
| Describe whether conclusions were supported by the evidence: |  | |  | | |  | |  | |
| A. Did the interpretation of findings address all of the concerns identified in Domains 1 to 4? | | |  | | |  | |  | |
|  | PY | | PN | | | Y | | Y | |
| B. Was the relevance of identified studies to the review's research question appropriately considered? | | |  | | |  | |  | |
|  | PY | | PY | | | Y | | Y | |
| C. Did the reviewers avoid emphasizing results on the basis of their statistical significance? |  | |  | | |  | |  | |
|  | PN | | PN | | | Y | | PN | |
| Risk of bias in the review RISK: LOW/HIGH/UNCLEAR |  | |  | | |  | |  | |
|  | UNCLEAR | | UNCLEAR | | | LOW | | UNCLEAR | |
| Rationale for risk: | how the data is presented | |  | | |  | | BECAUSE THEY STATE THERE IS MODERATE TO HIGH EVIDENCE FOR SYMPATHETIC BUT THE RESULTS STATE LOW/VERY LOW EVIDENCE | |
|  |  | |  | | |  | |  | |
| **Phase 2: Identifying concerns with the review process** |  | |  | | |  | |  |  |
| **DOMAIN 1: STUDY ELIGIBILITY CRITERIA** | Picchiottino 2019 | | Rechberger 2019 | | | Wirth 2019 | |  |  |
| 1.1 Did the review adhere to pre-defined objectives and eligibility criteria? | Y | | PY | | | PY | |  |  |
| 1.2 Were the eligibility criteria appropriate for the review question? | Y | | PY | | | PY | |  |  |
| 1.3 Were eligibility criteria unambiguous? | Y | | PY | | | PY | |  |  |
| 1.4 Were any restrictions in eligibility criteria based on study characteristics appropriate (e.g. date, sample size, study quality,outcomes measured)? | Y | | PY | | | PY | |  |  |
| 1.5 Were any restrictions in eligibility criteria based on sources of information appropriate (e.g. publication status or format, language, availability of data)? | PY | | PY | | | PY | |  |  |
| Concerns regarding specification of study eligibility criteria | LOW | | LOW | | | LOW | |  |  |
| Rationale for concern: |  | | no protocol | | | NO PROTOCOL | |  |  |
| **DOMAIN 2: IDENTIFICATION AND SELECTION OF STUDIES** |  | |  | | |  | |  |  |
| 2.1 Did the search include an appropriate range of databases/electronic sources for published and unpublished reports? | Y | | y | | | Y | |  |  |
| 2.2 Were methods additional to database searching used to identify relevant reports? | Y | | Y | | | Y | |  |  |
|  |  | |  | | |  | |  |  |
| 2.3 Were the terms and structure of the search strategy likely to retrieve as many eligible studies as possible? | Y | | Y | | | Y | |  |  |
|  |  | |  | | |  | |  |  |
| 2.4 Were restrictions based on date, publication format, or language I appropriate? | PY | | PY | | | PY | |  |  |
|  |  | |  | | |  | |  |  |
| 2.5 Were efforts made to minimise error in selection of studies? |  | |  | | |  | |  |  |
|  | Y | | PN | | | PY | |  |  |
| Concerns regarding methods used to identify and/or select studies LOW/HIGH/UNCLEAR |  | |  | | |  | |  |  |
|  | LOW | | unclear | | | LOW | |  |  |
| Rationale for concern: |  | | one reviewer | | |  | |  |  |
|  |  | |  | | |  | |  |  |
| **DOMAIN 3: DATA COLLECTION AND STUDY APPRAISAL** |  | |  | | |  | |  |  |
| 3.1 Were efforts made to minimise error in data collection? | Y | | PN | | | PY | |  |  |
| 3.2 Were sufficient study characteristics available for both review authors and readers to be able to interpret the results? | Y | | PY | | | PY | |  |  |
| 3.3 Were all relevant study results collected for use in the synthesis? | Y | | PY | | | PY | |  |  |
| 3.4 Was risk of bias (or methodological quality) formally assessed using appropriate criteria? | Y | | PY | | | PY | |  |  |
| 3.5 Were efforts made to minimise error in risk of bias assessment? | Y | | PN | | | PY | |  |  |
| Concerns regarding methods used to collect data and appraise studies LOW/HIGH/UNCLEAR | LOW | | unclear | | | LOW | |  |  |
| Rationale for concern: |  | | one reviewer | | |  | |  |  |
| **DOMAIN 4: SYNTHESIS AND FINDINGS** |  | |  | | |  | |  |  |
| 4.1 Did the synthesis include all studies that it should? | Y | | Y | | | Y | |  |  |
| 4.2 Were all pre-defined analyses reported or departures explained? | Y | | PN | | | PN | |  |  |
| 4.3 Was the synthesis appropriate given the nature and similarity in the research questions, study designs and outcomes across included studies? | Y | | PY | | |  | |  |  |
| 4.4 Was between-study variation (heterogeneity) minimal or addressed in the synthesis? | Y | | N | | | N | |  |  |
| 4.5 Were the findings robust, e.g. as demonstrated through funnel plot or sensitivity analyses? | PY | | N | | | N | |  |  |
| 4.6 Were biases in primary studies minimal or addressed in the synthesis? | PY | | Y | | | Y | |  |  |
| Concerns regarding methods used to collect data and appraise studies | LOW | | unclear | | | UNCLEAR | |  |  |
| Rationale for concern: |  | | STADISTICAL ANALYSIS POOR DESCRIBED | | | STADISTICAL ANALYSIS POOR DESCRIBED | |  |  |
| **Phase 3: Judging risk of bias** |  | |  | | |  | |  |  |
| Summarize the concerns identified during the Phase 2 assessment: |  | |  | | |  | |  |  |
| 1. Concerns regarding specification of study eligibility criteria |  | |  | | |  | |  |  |
|  | LOW | | LOW | | | LOW | |  |  |
| 2. Concerns regarding methods used to identify and/or select studies |  | |  | | |  | |  |  |
|  | LOW | | UNCLEAR | | | LOW | |  |  |
| 3. Concerns regarding methods used to collect data and appraise studies |  | |  | | |  | |  |  |
|  | LOW | | UNCLEAR | | | LOW | |  |  |
| 4. Concerns regarding the synthesis and findings |  | |  | | |  | |  |  |
|  | LOW | | UNCLEAR | | | UNCLEAR | |  |  |
| **RISK OF BIAS IN THE REVIEW** |  | |  | | |  | |  |  |
| Describe whether conclusions were supported by the evidence: |  | |  | | |  | |  |  |
| A. Did the interpretation of findings address all of the concerns identified in Domains 1 to 4? | | |  | | |  | |  |  |
|  | Y | | PY | | | PY | |  |  |
| B. Was the relevance of identified studies to the review's research question appropriately considered? | | |  | | |  | |  |  |
|  | Y | | Y | | | Y | |  |  |
| C. Did the reviewers avoid emphasizing results on the basis of their statistical significance? |  | |  | | |  | |  |  |
|  | PY | | PY | | | PY | |  |  |
| Risk of bias in the review RISK: LOW/HIGH/UNCLEAR |  | |  | | |  | |  |  |
|  | LOW | | UNCLEAR | | | LOW | |  |  |
| Rationale for risk: |  | |  | | |  | |  |  |
|  |  | |  | | |  | |  |  |
