# Supplementary material for: Do manual therapies have a specific autonomic effect? An overview of systematic reviews
Source: PLoS One. 2021 Dec 2;16(12):e0260642. doi: 10.1371/journal.pone.0260642 (PMC8638932; doi:10.1371/journal.pone.0260642)
Supplement: S4 Table — (DOCX) [file pone.0260642.s005.docx]

S4 Table. Concordance between overlapping of included reviews and summary of findings



|  | Schmid et al. 2008 | Hegedus et al. 2011 | Chu et al. 2014 | Kingston et al. 2014 | Lascurain et al. 2016 | Amoroso Borges et al. 2017 | Galíndez et al. 2017 | Araujo et al. 2018 | Navarro Santana et al. 2019 | Picchiottino et al. 2019 | Rechberger et al. 2019 |
| --- | --- | --- | --- | --- | --- | --- | --- | --- | --- | --- | --- |
| Hegedus et al. 2011 |  |  |  |  |  |  |  |  |  |  |  |
| Chu et al. 2014 |  |  |  |  |  |  |  |  |  |  |  |
| Kingston et al. 2014 |  |  |  |  |  |  |  |  |  |  |  |
| Lascurain et al. 2016 |  |  |  |  |  |  |  |  |  |  |  |
| Amoroso Borges et al. 2017 | NO | NO | NO | NO | NO |  |  |  |  |  |  |
| Galíndez et al 2017 | NO | NO | NO | NO | NO | NO |  |  |  |  |  |
| Araujo et al. 2018 |  |  |  |  |  |  | NO |  |  |  |  |
| Navarro Santana et al. 2019 |  |  |  |  |  | NO | NO |  |  |  |  |
| Picchiottino et al 2019 |  |  |  |  |  |  | NO |  |  |  |  |
| Rechberger et al. 2019 |  |  |  |  |  |  | NO |  |  |  |  |
| Wirth et al. 2019 | NO | NO | NO | NO | NO |  |  |  |  |  |  |
