# Supplementary material for: Do manual therapies have a specific autonomic effect? An overview of systematic reviews
Source: PLoS One. 2021 Dec 2;16(12):e0260642. doi: 10.1371/journal.pone.0260642 (PMC8638932; doi:10.1371/journal.pone.0260642)
Supplement: S5 Table — (DOCX) [file pone.0260642.s006.docx]

| **S5 Table. Agreements and discrepancies among reviews rating the same article** | | | | | | | | | | | | |
| --- | --- | --- | --- | --- | --- | --- | --- | --- | --- | --- | --- | --- |
| **Primary studies** | **Systematic Reviews** | | | | | | | | | | | |
|  | **Schmid 2008** | **Hegedus 2011** | **Chu 2014** | **Kingston 2014** | **Lascurain 2016** | **Amoroso-Borges 2017** | **Galíndez-Ibar 2017** | **Araujo 2018** | **Navarro-Santana 2019** | **Picchiotino 2019** | **Rechberger 2019** | **Wirth 2019** |
| Araujo et al 2018 |  |  |  |  |  |  |  |  |  | LowRoB |  |  |
| Arroyo-Morales et al., 2008 |  |  |  |  |  | good |  |  |  |  |  |  |
| Bakris et al. |  |  |  |  |  |  | LowRoB |  |  |  |  |  |
| Bowler et al |  |  |  |  |  |  |  |  |  | UnclearRoB |  |  |
| Budgell and Hirano (2001) |  |  |  |  |  | fair |  | poor |  | HighRoB | Moderate |  |
| Budgell and Polus(2006) |  |  |  |  |  | good |  | poor |  | HighRoB |  | Moderate |
| Cardoso de Mello et al |  |  |  |  |  |  |  |  |  |  | Moderate |  |
| Castro-Sanchez et al., 2010 |  |  |  |  |  | good |  |  |  |  |  |  |
| Chiu and Wright (1996) | high | High | good | good |  |  |  | fair | Unclear RoB |  | Limited |  |
| Chiu and Wright (1998) | high | Moderate |  |  |  |  |  |  | LowROB |  |  |  |
| Cleland |  |  |  |  |  |  |  |  | UnclearROB |  |  |  |
| Curi et al |  |  |  |  |  |  |  |  |  |  | Moderate |  |
| Da Silva et al |  |  |  |  |  |  |  |  |  |  |  | Moderate |
| Delaney, 2002 |  |  |  |  |  | good |  |  |  |  |  |  |
| Fornari |  |  |  |  |  |  |  |  |  |  | Moderate |  |
| Fukuda et al |  |  |  |  |  |  |  |  |  |  | Limited |  |
| Gibbons et al |  |  |  |  |  |  |  |  |  |  | Limited |  |
| Giles et al |  |  |  |  |  |  |  |  |  |  | Limited |  |
| Goertz et al 2016a |  |  |  |  |  |  |  |  |  |  |  | Moderate |
| Gosling et al (2005) |  |  |  |  |  |  |  | fair |  |  |  |  |
| Henderson et al |  | Moderate |  |  |  |  |  |  |  | HighRoB | Moderate |  |
| Henley et al., 2008 |  |  |  |  |  | good |  |  |  |  |  |  |
| Jowsey and Perry (2010) |  | High | good | good |  |  |  | good | UnlearRoB | UnclearRoB | Moderate |  |
| Knutson |  |  |  |  |  |  | LowRoB |  |  |  |  |  |
| Kovanur et al |  |  |  |  |  |  |  |  |  |  |  | Moderate |
| La Touche et al (2013) |  |  | excelent |  | LowRoB |  |  | excelent | LowRoB | UnclearRoB | Strong |  |
| McGuiness et al (1997) | high |  |  | good |  |  |  | fair |  | UnclearRoB |  |  |
| Milnes and Moran et al |  |  |  |  |  |  |  |  |  |  | Limited |  |
| Morgan et al |  |  |  |  |  |  |  |  |  |  |  | Moderate |
| Moulson et al (2006) |  | Moderate | good |  |  |  |  | good | UnclearRoB | UnclearRoB |  |  |
| Moutzouri et al (2012) |  |  |  |  |  |  |  | good | UncleraRoB | UnclearRoB |  |  |
| Paungmali et al |  |  |  |  |  |  |  |  | UncleraRoB | UnclearRoB |  |  |
| Perry and Green (2008) |  | High |  | good |  |  |  |  |  | UncleraRoB |  |  |
| Perry and Green (2011) |  |  |  |  |  |  |  | excelent | LowRoB |  |  |  |
| Perry et al (2011) |  |  |  |  |  | fair |  | good |  |  |  | Moderate |
| Perry et al (2015) |  |  |  |  |  |  |  |  |  |  |  | Moderate |
| Petersen et al. (1993) | high | Moderate | good | good |  |  |  |  | LowRoB | UnclearRoB | Limited |  |
| Piekarz and Perry |  |  |  |  |  |  |  |  | LowRoB | LowRoB |  |  |
| Puhl et al (2012) |  |  |  |  |  |  |  | good |  | UnclearRoB |  |  |
| Purdy et al |  |  |  |  |  |  |  |  |  |  | Moderate |  |
| Roy et al., 2009 |  |  |  |  |  | good |  |  |  | UnclearRoB | Moderate | Moderate |
| Ruffini et al |  |  |  |  |  |  |  |  |  |  | Strong |  |
| Sampath et al |  |  |  |  |  |  |  |  |  | UnclearRoB |  |  |
| Scoppa et al |  |  |  |  |  |  |  |  |  |  | Limited |  |
| Shi et al |  |  |  |  |  |  |  |  |  |  | Strong |  |
| Sillevis et al (2010 |  |  |  |  |  |  |  | fair |  | UnclearRoB |  | Moderate |
| Simon |  |  |  |  |  |  |  |  | UnclearRoB | UnclearRoB |  |  |
| Slater et al (1994) |  | Moderate |  |  |  |  |  | fiar | LowRoB | UnclearRoB |  |  |
| Sterling et al (2001) | high |  |  | excelent | LowRoB |  |  | good | LowRoB | UnclearRoB |  |  |
| Tsirakis |  |  |  |  |  |  |  |  | LowRoB | UnclearRoB |  |  |
| Vicenzino et al (1994) | high |  | excelent |  |  |  |  | fair | UnclearRoB | UnclearRoB |  |  |
| Vicenzino et al (1995) | high |  |  |  |  |  |  |  |  |  |  |  |
| Vicenzino et al (1998)a | high |  |  | good | ModerateRoB |  |  | fair | LowRoB | UnclearRoB |  |  |
| Vicenzino et al (1998)b | high |  | excelent |  |  |  |  |  |  | UnclearRoB |  |  |
| Ward et al |  |  |  |  |  |  | LowRoB |  |  | UnclearRoB |  | Moderate |
| Welch, Boone, 2008 |  |  |  |  |  | poor |  |  |  |  | Limited |  |
| Win et al |  |  |  |  |  |  |  |  |  |  | Moderate | Moderate |
| Younes et al |  |  |  |  |  |  |  |  |  |  | Moderate |  |
| Yung et al 2014 |  |  |  |  |  |  |  |  |  | UnclearRoB |  |  |
| Yung et al 2017 |  |  |  |  |  |  |  |  |  | UnclearRoB |  |  |
| Zegarra-Parody |  |  |  |  |  |  |  |  | UnclearRoB | UnclearRoB |  |  |
| Zhang et al., 2006 |  |  |  |  |  | fair |  |  |  |  | Moderate |  |

|  | Cochrane: low, moderate, unclear, high risk of bias | |
| --- | --- | --- |
|  | GRADE: limited, moderate, strong evidence |  |
|  | PEDRO: poor quality, fair quality, good quality, 9excellet quality | |
|  | Down and Black: poor, limited, moderate, strong evidence |  |
|  | JADAD: high quality, moderate quality, low quality |  |
