# Supplementary material for: Do manual therapies have a specific autonomic effect? An overview of systematic reviews
Source: PLoS One. 2021 Dec 2;16(12):e0260642. doi: 10.1371/journal.pone.0260642 (PMC8638932; doi:10.1371/journal.pone.0260642)
Supplement: Attachment — Submitted filename: Response to reviewers.docx [file pone.0260642.s008.docx]

Response to reviewers 1/11/21

| Comments | Responses |
| --- | --- |
| Rev#2 | |
| **General remark** | Thank you for giving us the opportunity to keep improving the manuscript |
| **Abstract** | |
| Again, please stick to Journal guidelines. Maximum word count is 300 (but NOT 399!). Please revise carefully. Remember that your answer ("Thank you for this comment, Abstract has been shortened considerably") would not seem satisfying | Amended. The abstract counts 267 words. |
| Again, please adapt aims given with the Abstract section and the questions asked in the Introduction section. This reviewer has read your comments ("Thank you for this clarification, corrections have been amended in the Abstract page 2 lines 20-21"), but still there would be some need to polish this topic. | Amended. |
| Remember that with your conclusions, answers must be given exclusively to your questions | Amended |
| **Introduction** | |
| Please see comments given above. | Amended Page 4 lines 129-133 |
| **Materials and Methods** | |
| Heading must read "Materials and methods". Again, please stick to the Journal guidelines, and consult some recently published Plos One papers. "METHODS" would not seem acceptable. Remember that there will not be any thorough copy editing, so only flawless papers will be acceptable | Amended Page 6 line 154 |
| As a general recommendation, please stick to Journal style with ALL other aspects. For example, you repeatedly refer to "table 2", "table 3", "table 4", and so on. Again, please consult some recently published Plos One papers, there you will see that this must read "Table (1, 2, 3, or 4)". Same with other minor aspects, please revise carefully, and remember that submitting a flawless manuscript is considered the Authors' task, and that the typesetter will not be able to copy edit your draft. | Tables, figures and supplementary information has been adapted to the journal requirements from <https://journals.plos.org/plosone/s/tables>  <https://journals.plos.org/plosone/s/supporting-information>  <https://journals.plos.org/plosone/s/submission-guidelines>  See highlighted marks. |
| **Conclusions** | |
| Do not give a "summary" here. For example, "There is considerable research (...)" and "This overview summarized the information reported by 12 systematic reviews." are not considered conclusions. | Conclusions section has been amended. Page 25 |
| Again, with your Conclusions section, please stick exclusively to your revised aims. Do not simply repeat your results here. Instead, provide a reasonable and generalizable extension of your outcome. | Conclusions section has been amended. Page 25 |
| Same thoughts are valid for "Implications for research: (...)". These aspects surely would seem right, and should be transferable to the Discussion section. However (and again), those thoughts are not considered Conclusions. Again, please revise carefully. | Amended. Mentions to Implications for research have been moved to Discussion. |
| **References** | |
| - Again, revise for uniform formatting. Stick to Journal style. Provide doi and PMID numbers. Again, the Authors' response ("We apologise for this inconvenience and references have been amended as suggested.") would not seem satisfying  - Example would be: "Cheng L, Weir MD, Xu HH, Antonucci JM, Lin NJ, Lin-Gibson S, et al. Effect of amorphous calcium phosphate and silver nanocomposites on dental plaque microcosm biofilms. J Biomed Mater Res B Appl Biomater. 2012; 100(5): 1378–1386. <https://doi.org/10.1002/jbm.b.32709> PMID: 22566464" Revise thoroughly, but carefully. Remember that it is not the idea to re-re-review your submitted draft, and another re-submission not considered satisfying would lead to "outright rejection | Amended. |
